# Assessment of Sonication for Diagnosing Implant-Associated Infections in Spinal Surgery Routine Practice

**DOI:** 10.3390/microorganisms13081898

**Published:** 2025-08-14

**Authors:** Estibaliz Torrecilla-Sádaba, Santiago Gabardo, Ignacio Mahillo-Fernández, Pierre Ferrer Pomares, Félix Tome-Bermejo, Luis Álvarez-Galovich, Joaquín García-Cañete, Jaime Esteban, Charles Mengis

**Affiliations:** 1 Department of Clinical Microbiology, IIS-Fundacion Jimenez Díaz, Universidad Autónoma de Madrid, 28040 Madrid, Spain; estibaliz.torrecilla@quironsalud.es; 2 Spine Surgery Unit, Hospital Universitario Fundación Jiménez Díaz, Avda. Reyes Católicos, 2, 28040 Madrid, Spain; 3 Department of Epidemiology, IIS-Fundación Jiménez Díaz, Universidad Autónoma de Madrid, 28040 Madrid, Spain; 4 Spine Surgery Unit, Hospital Universitario General de Villalba, 28400 Collado Villalba, Spain; 5 Department of Internal Medicine, Hospital Universitario Fundación Jiménez Díaz, IIS-Fundación Jiménez Díaz, Universidad Autónoma de Madrid, 28040 Madrid, Spain; 6 CIBERINFEC-CIBER de Enfermedades Infecciosas, 28029 Madrid, Spain

**Keywords:** revision spine surgery, spine, surgical site infection, occult infection, sonication, biofilm

## Abstract

Infections following spinal surgery can result in potentially devastating complications. An accurate microbiological diagnosis is crucial for proper treatment. Sonication is a diagnostic method that can be beneficial in patients with acute or low-grade infections. This study aimed to assess the sensitivity and effectiveness of sonication as a method for diagnosing spinal implant infections in cases of both suspected and unsuspected infections during spinal surgical revision. We conducted a retrospective observational study that included all patients who underwent revision spinal surgery between March 2011 and October 2022. We collected the implants and surrounding tissues from these patients for microbiological analysis. The implant sonication was performed according to a previously published protocol. Patients were categorised into those undergoing surgical revision for suspected spinal implant infection (SSII) and those for non-suspected spinal implant infection (NSSII). We collected comprehensive patient data, including demographics, risk factors, Charlson Comorbidity Index (CCI), surgical details, microbiological findings, antibiotic regimens, and clinical outcomes. Sensitivity and specificity analyses were conducted on both sonicated and non-sonicated samples. A total of 158 patients met the inclusion criteria; 51 of them were diagnosed with infection during surgery revision. Patients with SSII had higher CCIs than those with NSSII. The sensitivity was significantly higher in sonicated samples (68.6%; 95% CI: 55.9–81.4%) than in non-sonicated samples (42%; 95% CI: 28.3–55.7%). The specificities were similar, with sonicated samples at 93.5% (95% CI: 88.8–98.1%) and non-sonicated samples at 99.05% (95% CI: 97.2–100.9%). Combining both methods resulted in sensitivity and specificity rates of 76% (95% CI: 64.2–87.8%) and 93.3% (95% CI: 88.2–98.1%), respectively. Methicillin-susceptible *Staphylococcus aureus* (MSSA) was common in SSII, whereas *Cutibacterium acnes* and coagulase-negative Staphylococcus (CNS) were predominant in NSSII. This study supports the routine use of implant sonication as a valuable supplementary method for peri-implant tissue cultures, especially for identifying low-grade spinal implant infections.

## 1. Background Context

A meta-analysis and systematic review by Zhou et al. included 27 studies with a total of 22.475 patients and 603 cases of spinal surgical site infections, reporting an overall incidence of 3.1% [1]. The incidence varied according to several factors: type of infection (1.4% for superficial vs. 1.7% for deep infections), patient population (higher rate of 13% in patients with neuromuscular scoliosis), spinal level (3.4% cervical, 3.7% thoracic, 2.7% lumbar), surgical approach (5% posterior vs. 2.3% anterior), instrumentation (4.4% with instrumentation vs. 1.4% without), and surgical invasiveness (1.5% in minimally invasive vs. 3.8% in open procedures). These differences may be due to heterogeneity in patients’ characteristics, surgical procedures or approaches, microbiological diagnostic techniques, and the absence of standardised guidelines for defining spinal implant infection and treatment protocol [2].

Infections can be categorised as acute or chronic, depending on when the symptoms appear, which leads to differences in their management.

Early acute infections are usually caused by aggressive microorganisms, such as *S. aureus*. They typically show classic signs of infection, including fever, issues with wound healing, and elevated inflammatory markers.

In contrast, chronic or low-grade infections, which appear later, are usually caused by less aggressive microorganisms such as *C. acnes* and *coagulase-negative Staphylococcus* (CNS). These infections may have no symptoms or nonspecific symptoms such as chronic pain, implant looseness, lack of bone healing, or wound dehiscence, with minimal changes in inflammatory markers [3,4]. Known as occult infection, they are challenging to diagnose clinically, due to their subtle or absent symptoms, and microbiologically because the causative organisms are slow-growing. They may require prolonged culture incubation or yield false-negative results [5,6]. Management strategies differ depending on the presentation; early-onset infections may be treated with debridement and implant retention, whereas late-onset infections usually require partial or complete removal of the implant [2,3].

Traditionally, spinal implant failure without clear signs of infection has been associated with mechanical overload and aseptic loosening. However, there is increasing recognition that low-virulence organisms may contribute to presumed aseptic failure [5,7]. *C. acnes* and CNS are the most isolated microorganisms responsible for such infections [5,6,8]. These bacteria can cause late-onset infections by forming biofilms on the surface of implants [4,5]. Studies have shown that the presence of biofilm can lead to implant failure, causing local reactions and resulting in separation at the bone–device interface, resulting in an indolent infection [5].

A biofilm is a structured aggregation of bacteria encased in a self-produced matrix of extracellular polysaccharides that adheres to a surface. Materials commonly used for spinal implants are susceptible to colonisation by biofilm-forming bacteria. Biofilm development occurs in four stages: initial bacterial adhesion to the foreign material, cell aggregation, biofilm maturation, and detachment of cells. Once the biofilm is established, bacteria exhibit increased resistance to antibiotics and the host immune system. Although the exact mechanisms are not fully understood, this resistance may be related to the presence of dormant or slow-growing bacterial cells within the biofilm structure [4]. At this stage, conventional methods often fail to detect bacteria, as the organisms are embedded within the biofilm and not readily accessible [4,8]. To enhance the sensitivity of diagnosis, sonication has emerged as a valuable technique by applying high-frequency ultrasound to mechanically dislodge bacteria from the implant and detect bacteria from biofilms by increasing the number of culturable cells [8].

Acute spinal implant infections are typically diagnosed based on clinical, radiological, and laboratory parameters. However, in chronic cases, these parameters may show minimal changes or may even be absent, which can make diagnosis challenging [9]. Sonication has been increasingly used to detect microorganisms in both types of infections, particularly hidden infections, where it may be the only method of diagnosing the infection [2,7].

This study aimed to evaluate the role of sonication of explanted spinal implants in enhancing microbial detection and diagnostic sensitivity, with a focus on its ability to increase the yield of positive cultures, particularly in low-grade or occult infections.

## 2. Materials and Methods

We carried out a retrospective observational study at our centre. The study included patients aged 18 years and older who underwent spinal revision surgery at our institution between March 2011 and October 2022. All patients in the study underwent microbiological cultures on their implants (referred to in this article as sonicated samples) and the surrounding peri-implant tissues (referred to as non-sonicated samples). Additionally, we conducted a minimum one-year follow-up, during which we collected all patient data from their medical records. Patients under 18 years of age, those without implants or peri-implant tissues, those treated at another hospital, those with incomplete medical records, or those with a follow-up of less than one year were excluded from the study.

A multidisciplinary team consisting of microbiologists, internists, and traumatologists collaborated to establish an infection diagnosis. The microbiological, clinical, and radiological data of each patient were carefully evaluated. Spinal implant infection was suspected if patients met at least one of the following criteria: wound dehiscence, material exposure, compatible imaging findings, or presence of a fistula.

Patients were categorised based on two criteria: the onset of infection and meeting the criteria for infection during spinal surgery revision. For the onset, early-onset infection occurred when the time between the surgical revision and the onset was less than three months. In contrast, late-onset infection occurred more than three months after surgical revision [3,10]. Patients meeting the criteria for infection were classified as having suspected spinal implant infections (SSIIs), whereas those not meeting this criterion were classified as having no suspected spinal implant infections (NSSIIs).

### 2.1. Microbiological Procedures

Samples were obtained from patients who underwent revision spinal surgery in the operating rooms of the hospital and were then sent to the microbiology laboratory for processing. If immediate processing was not possible, samples were refrigerated at 4 °C for up to 24 h. All implants underwent sonication according to the hospital’s protocol [11]. Briefly, each implant was placed in a hermetically sealed sterile plastic container with 50 mL of sterile phosphate buffer (pH 6.8), then sonicated for 5 min using a low-power bath sonicator (JP Selecta, Barcelona, Spain). The sonicated fluid was centrifuged at 3000 g for 20 min, and 10 µL of the supernatant weres inoculated onto each plate. The minimal number of CFU/mL that can be detected by this methodology is 100 CFU/mL. At the same time, peri-implant tissues were processed by mechanical grinding.

Implant and peri-implant tissues were cultured on the same media, including Tryptic soy-5% sheep blood agar, chocolate agar, MacConkey agar, and Schaedler-5% sheep blood agar for anaerobic cultures (all from bioMérieux, Marcy l’Etoile, France). Additionally, mycological media such as Sabouraud Chloramphenicol agar and Sabouraud Chloramphenicol Actidione agar (bioMérieux) were used for fungal culture. Mycobacterial media, including Löwestein-Jensen and Coletsos solid media, as well as mycobacterial liquid cultures using automated systems such as bioMérieux BacT/Alert and BD BACTEC MGIT 320, were used for peri-implant biopsies when mycobacterial infection was suspected.

The incubation period was seven days for bacteriological cultures (extended to 14 days if cultures were negative and clinical suspicion of infection was high), four weeks for mycological cultures, and eight weeks for mycobacterial cultures.

In cases where there was unexpected growth, possible contamination, or suspicion of low-grade infection, diagnosis was assessed using a combination of the clinical and microbiological criteria. Instead of clinical considerations, we considered the bacterial load on the plate, as well as the number of distinct peri-implant tissues or implant samples exhibiting the same microbiological growth, following the criteria established by Atkins et al. [12] but including the implant sonicate as an additional sample. We considered the varying virulence of the microorganisms involved, distinguishing between low-virulence bacteria commonly found in the cutaneous flora and highly virulent pathogens. Cases with high colony counts, growth of a virulent pathogen (e.g., Gram-negative bacilli or Staphylococcus aureus), and/or growth in more than one sample were considered more likely to represent actual infection rather than contamination. Additionally, even when colony counts were low (including zero), the isolation of a virulent microorganism in two or more separate samples was also considered indicative of infection.

### 2.2. Clinical Data

The ethics committee of our institution approved this study. We gathered baseline data from patients in our institution’s medical records. These data included demographic information such as age, sex, and body mass index (BMI) as well as risk factors such as high blood pressure (HBP), diabetes, smoking, dyslipidemia, oncological disease, autoimmune disease, and anticoagulation disease. We also collected data on the Charlson Comorbidity Index (CCI), number and location of fused levels, number of surgeries, diagnostic results during revision surgery, microbiological findings, and antibiotic treatment. We used frequency and percentage to describe most variables, while CCI was presented as mean and standard deviation.

### 2.3. Statistical Analysis

Logistic regression models were used to compare the variables. For CCI, an odds ratio (OR) greater than one indicates that a higher CCI is associated with a higher probability of having the analysed condition. For the other variables, the first category was the reference category, and the regression model calculated an OR for each remaining category, comparing them to the reference category. An OR > 1 indicates a higher probability of having the analysed condition in that category compared to the reference category. Conversely, an OR < 1 indicates the opposite.

Fisher’s exact test for comparison proportions was used to compare the sensitivity and specificity of peri-implant tissues with those of sonicated implants. Statistical significance was set at *p* < 0.05.

## 3. Results

### 3.1. Description of the Study Population

A total of 158 patients met the inclusion criteria and were included in this study, comprising 53.8% males and 46.2% females, with a mean age of 63.15 ± 14.83 years (range: 20–89 years). All patients had previously undergone arthrodesis, primarily for degenerative conditions (N = 114; 72.2%), followed by spinal deformity (N = 31; 19.6%), fractures (n = 10; 6.3%), and tumours (n = 3; 1.9%). This index surgery was primarily performed in the lumbar (n = 123, 77.8%) and dorsolumbar (n = 25, 15.8%) regions, with cervical (n = 5, 3.2%), dorsocervical (n = 2, 1.3%), and dorsal (n = 3, 1.9%) surgeries being less common. The levels involved in this primary surgery were one in 59 patients (37.3%), two in 47 patients (29.7%) and three or more in 52 patients (32.9%).

During spinal surgery revision, 40 patients (29.7%) met infection criteria and were classified in the SSII group, while 118 patients (74.7%) did not and were in the NSSII group. The patients in the NSSII group, without suspected infection, were mainly reoperated on because they suffered pseudoarthrosis (N = 46; 38.9%) or proximal junctional kyphosis (N = 41; 34.7%). Other causes of reoperation included postoperative pain (N = 18, 11.5%), implant failure (N = 10, 8.4%), and sagittal imbalance (N = 3, 2.6%).

The time between the initial intervention and revision implant surgery was as follows: Less than three months for 25 patients (15.8%) (14 SSII group vs. 11 NSSII group; *p*-value = 0.0001), three months to one year for 29 patients (18.3%) (10 SSII group vs. 19 NSSII group; *p*-value = 0.2090), one–three years for 37 patients (23.4%) (4 SSII group vs. 33 NSSII group; *p*-value = 0.0204), three–five years for 23 patients (14.5%) (7 SSII group vs. 16 NSSII group; *p*-value = 0.5414), five–ten years for 22 patients (13.9%) (1 SSII group vs. 21 NSSII group; *p*-value = 0.0157), and more than ten years for 22 patients (13.9%) (4 SSII group vs. 18 NSSII group; *p*-value = 0.4068).

When comparing patients included in the SSII group to those in the NSSII group, there were statistically significant differences in CCI and smoking habits. The CCI was higher in the SSII group (3.30 ± 1.96 vs. 2.42 ± 1.97; OR 1.25, *p*-value = 0.016). Conversely, the number of smokers was higher in the NSSII group (49 vs. 8; OR, 0.35; *p* = 0.017) (Table 1).

For patients who met the criteria of infection (SSII group), the diagnosis included patients with both positive (N = 23) and negative microbial culture (N = 8) results, investigating whether the cause of negative cultures could be due to previous antibiotic use. For patients who did not meet the criteria of infection (NSSII group), the diagnosis was based on unexpected positive cultures that raised suspicion of an occult infection (n = 16) or because the patients did not heal even though the cultures were negative (n = 4). All 31 cases in the SSII group and all 20 cases in the NSSII group were treated with antibiotics. However, seven microbiological findings in the NSSII group and 1 in the SSII group were considered contaminants, and those patients did not receive antibiotic treatment.

Of the infected cases, 14 (27.5%) (11 SSII group vs. 2 NSSII group; *p*-value = 0.0415) had an early-onset infection (three months or less), and 37 (72.5%) (20 SSII group vs. 18 NSSII group; *p*-value = 0.0415) had a late-onset infection (more than three months).

In our series, there was only one case of recurrence with the same microorganism (*C. acnes*), which occurred in the NSSII group.

Regarding clinical progression, 16 patients in the SSII group and 5 in the NSSII group required additional surgeries (*p*-value = 0.0594).

### 3.2. Sensitivity and Specificity of Sonication

The sensitivities of sonicated (prosthesis samples) and non-sonicated samples (surrounding tissues) were 68.6% (95% confidence interval (CI): 55.9–81.4%) and 42% (95% CI: 28.3–55.7%), respectively (*p* = 0.009), with a significantly higher sensitivity in sonicated samples compared to non-sonicated samples. Specifically, 17 patients diagnosed with infection had a positive culture in sonicated samples and a negative culture in non-sonicated samples, whereas four patients showed a positive culture in non-sonicated samples and a negative culture in sonicated samples.

The specificities of sonicated and non-sonicated samples were 93.5% (95% CI: 88.8–98.1%) and 99.05% (95% CI: 97.2–100.9%), respectively (*p* = 0.065), showing no statistically significant differences. Contaminants were more frequent in sonicated samples (n = 7) than in non-sonicated samples (n = 1), all of which belonged to the NSSII group.

Combining both methods increased the sensitivity from 42% in non-sonicated samples and 68.6% in sonicated samples to 76% (95% CI: 64.2– 87.8%), while the specificity remained similar to that of the sonicated method at 93.3% (95% CI: 88.2– 98.1%) (Table 2).

### 3.3. Isolated Microorganisms

The most frequently isolated microorganisms in NSSII were *C. acnes* (n = 5; 31.3%) and CNS (n = 5; 31.3%), whereas in SSII, it was methicillin-susceptible *S. aureus* (MSSA) (n = 6; 26.1%), followed by different species of *Enterobacteriaceae* (n = 4, 17.4%).

In both groups, the number of microorganisms isolated by sonication was higher than that in the non-sonicated samples, with statistically significant differences observed in the NSSII group (*p*-value = 0.0005) but not in the SSII group (*p*-value = 0.2) (Table 3).

## 4. Discussion

Several recent studies have examined the use of sonication in patients with spinal implant infections. These studies differentiated between its use in cases with presumed aseptic failure [8,13] and cases with signs or symptoms of infection [14,15,16]. To the best of our knowledge, this is the first study to combine unexpected positive cultures in presumed aseptic failures with cases meeting the criteria for infections, considering spinal implant infection as a combination of both. It is well known that spinal infections often present with nonspecific symptoms, especially in late-onset infections where symptoms may be absent, and laboratory parameters and radiological findings may not be affected [2,3,8,9,13,17].

The only statistically significant difference observed between patients in both groups was a higher CCI in the SSII group than in the NSSI group. CCI is a well-established risk factor for spinal implant infections [3,17,18].

Our study shows that sonication of retrieved implants is significantly more sensitive than biopsy culture methods in detecting spinal implant infection samples without a significant loss in specificity, compared to similar studies. However, our study reported a lower sensitivity than previous studies [14,15,16], possibly due to the inclusion of presumed aseptic failures. Unexpected positive cultures in such infections may indicate low-grade infections caused by low-virulence microorganisms, which are more challenging to diagnose owing to their subtle symptoms, limited laboratory parameters, or radiologic findings [2,3,8,9,13].

While the sonication method showed higher sensitivity and detected more bacteria than the non-sonicated methods, our study found that some bacteria were only detected using the non-sonicated methods. Therefore, it is important to include sonication as an additional step in the traditional biopsy culture. The combination of both methods led to higher specificity and sensitivity, which aligns with the findings of Bürger et al. [14].

The additional centrifugation step added to the sonication process at our institution has proven to be effective in treating other implant-related infections. This method could be beneficial for patients with low bacterial loads, as it allows for sample concentration [19,20]. However, the addition of this new manipulation step to the sample may lead to increased culture contamination. Therefore, a multidisciplinary team must interpret these results carefully, considering all aspects of the patient’s condition.

Sonication notably isolated more microorganisms than the non-sonicated methods, particularly in cases without suspected infection (NSSII group). This underscores the importance of detecting infections in presumed aseptic failures, which are usually challenging to diagnose and manage [2]. Nonetheless, sonicated methods involve more sample manipulation steps than traditional culture methods, and may result in increased bacterial contamination, a matter of ongoing controversy [8,13].

Despite the advantages of sonication, such as its ability to detect low-virulence microorganisms implicated in spinal implant infections, it also introduces complexity and increases contamination risks. However, Rondaan et al. [21] recently published a European multicenter case–control study in which patients with positive growth in sonicated fluid only were treated (>50 CFU/mL). At the same time, patients not treated with negative cultures in all samples served as controls. Patients were followed until failure occurred or for at least one year (mean 1158 days; range 883–1590 days). The study showed that the infection rate during follow-up was nearly twice as high in cases compared to controls, highlighting the clinical relevance of sonication. Furthermore, Akgün et al. [9] demonstrated that low-virulence microorganisms might not significantly alter conventional diagnostic parameters in delayed spinal implant infections. Consequently, unexpected positive cultures, identified primarily by sonication, are common in these infections. Given the significant implication of these low-virulence microorganisms in spinal implant infections, sonication has emerged as a crucial diagnostic tool, complementing peri-implant tissue culturing and other sampling methods, as it alone may not identify all causative microorganisms, as demonstrated in our study.

The microorganisms most frequently isolated in our study (*C. acnes*, CNS, and *S. aureus*) were consistent with those reported in similar studies [8,13,14,15,16]. In patients undergoing revisional surgery without suspected spinal infection (NSSII group), we mainly found low-virulence microorganisms, such as *C. acnes* and CNS. These bacteria tend to produce unexpected positive cultures because they can form biofilms. This can lead to implant failure without an evident septic infection. Typically, biofilm formation leads to latent infection, which primarily presents with chronic or no symptoms until device dysfunction occurs [5,8].

Conversely, in cases where infection was suspected during revision surgery (SSII group), we frequently identified highly virulent microorganisms such as *S. aureus* and Enterobacterales. These microorganisms commonly lead to infections with signs and symptoms, usually presenting with an early onset. This observation is consistent with the study by Nieto et al. [22], who identified the same bacteria as the principal cause of spinal implant infection. This correlation may be because the study population primarily consisted of patients exhibiting signs and symptoms of infection with an acute presentation.

One notable strength of this study was its pragmatic approach, closely mirroring real-life scenarios in a tertiary hospital setting. Another significant strength is the large sample size, which facilitates robust data collection regarding the sensitivity and specificity of the sonication technique. In addition, the comprehensive extraction of diverse patient data from medical charts enables a thorough description of the study population.

However, this study has several limitations that warrant consideration. This retrospective design introduced inherent bias. Furthermore, despite the large sample size, the low number of infection cases impeded definitive conclusions. The absence of information on prior antibiotic use could influence the culture sensitivity. Other additional challenges include variations in surgical procedures, heterogeneous patient populations, diverse indications for primary and revision surgeries, and uneven case distributions over time. Moreover, the lack of a standardised definition for spinal implant infection necessitated reliance on hospital protocols.

In conclusion, this study supports the routine use of implant sonication as a valuable adjunct method to peri-implant tissue culture, particularly in identifying low-grade spinal implant infections. These findings emphasise the importance of integrating sonication into diagnostic protocols to optimise patient management, care, and outcomes.

## Figures and Tables

**Table 1:** Demographic characteristic comparison between suspected and no suspected spinal implant infection.

Variable	NSSII (n = 118)	SSII (n = 40)	OR (95% CI)	*p*-Value
**CCI**	2.42 ± 1.97	3.30 ± 1.96	1.25 (1.04, 1.50)	0.016
**Age**				
18–39	7 (77.8%)	2 (22.2%)	Reference	
40–64	61 (87.1%)	9 (12.9%)	0.52 (0.10, 3.82)	0.451
65–74	24 (64.9%)	13 (35.1%)	1.90 (0.39, 14.0)	0.464
>75	26 (61.9%)	16 (38.1%)	2.15 (0.45, 15.7)	0.374
**Gender**				
Female	60 (70.6%)	25 (29.4%)	Reference	
Male	58 (79.5%)	15 (20.5%)	0.62 (0.29, 1.28)	0.203
**BMI**				
≤30	73 (71.6%)	29 (28.4%)	Reference	
>30	38 (84.4%)	7 (15.6%)	0.46 (0.17, 1.11)	0.099
**HBP**				
No	70 (77.8%)	20 (22.2%)	Reference	
Yes	48 (70.6%)	20 (29.4%)	1.46 (0.71, 3.01)	0.305
**Diabetes**				
No	97 (75.2%)	32 (24.8%)	Reference	
Yes	21 (72.4%)	8 (27.6%)	1.15 (0.44, 2.78)	0.756
**Smoker**				
No	69 (68.3%)	32 (31.7%)	Reference	
Yes	49 (86.0%)	8 (14.0%)	0.35 (0.14, 0.80)	0.017
**Dyslipidemia**				
No	86 (78.2%)	24 (21.8%)	Reference	
Yes	32 (66.7%)	16 (33.3%)	1.79 (0.84, 3.79)	0.128
**Oncologica** **l disease**				
No	108 (76.6%)	33 (23.4%)	Reference	
Yes	10 (58.8%)	7 (41.2%)	2.29 (0.78, 6.45)	0.119
**Autoimmune disease**				
No	111 (75.0%)	37 (25.0%)	Reference	
Yes	7 (70.0%)	3 (30.0%)	1.29 (0.27, 4.89)	0.725
**Anticoagulation**				
No	99 (76.7%)	30 (23.3%)	Reference	
Yes	19 (65.5%)	10 (34.5%)	1.74 (0.71, 4.08)	0.212
**Surgical location**				
Cervical	4 (80.0%)	1 (20.0%)	Reference	
Dorsocervical	1 (50.0%)	1 (50.0%)	4.00 (0.10, 221)	0.442
Dorsal	1 (33.3%)	2 (66.7%)	8.00 (0.38, 383)	0.210
Dorsolumbar	14 (56.0%)	11 (44.0%)	3.14 (0.39, 66.3)	0.335
Lumbar	98 (79.7%)	25 (20.3%)	1.02 (0.14, 20.4)	0.986

OR, Odds Ratio; NSSII: no suspected spinal implant infection; SSII: suspected spinal implant infection; CCI: Charlson Comorbidity Index; BMI: Body Mass Index; HBP: High Blood Pressure.

**Table 2:** Sensitivities and specificities of sonicated and non-sonicated samples.

	Sensitivity	Specificity
Sonicated samples	68.6% (95% CI: 55.9–81.4%)	93.5% (95% CI: 88.8–98.1%)
Non-sonicated samples	42% (95% CI: 28.3–55.7%)	99.05% (95% CI: 97.2–100.9%)
*p*-value	0.009	0.065

**Table 3:** Etiological distribution of isolated microorganisms.

Infection Diagnosis (39/51)				
Microorganism	NSSII (16/20)		SSII (23/31)	
	Sonicated (15/20)	Non-Sonicated (4/20)	Sonicated (20/31)	Non-Sonicated (15/30)
*Cutibacterium acnes*	5	1	2	1
CNS	5	0	2	1
*Pseudomonas aeruginosa*	1	2	0	0
*Streptococcus mitis/oralis*	1	1	0	0
Polymicrobial infections	2	0	3	3
*Escherichia coli*	1	0	3	1
MSSA	0	0	5	4
*Corynebacterium afermentans*	0	0	1	0
*Enterococcus faecalis*	0	0	3	3
*Morganella morganii*	0	0	1	0
*Acinetobacter baumanii*	0	0	0	1
*Corynebacterium urealyticum*	0	0	0	1
Contaminants (7/107)				
Microorganism	NSSII (7/98)		SSII (0/9)	
	Sonicated (7/98)	Non-sonicated (1/97)	Sonicated (0/9)	Non-sonicated (0/8)
*Cutibacterium acnes*	2	0	0	0
*Staphylococcus epidermidis*	2	0	0	0
*Candida parapsilosis*	1	1	0	0
*Corynebacterium* spp.	1	0	0	0
*Enterococcus faecium*	1	0	0	0

NSSI, no suspected spinal implant infection; SSII, suspected spinal implant infection; CNS, coagulase-negative *Staphylococcus*; MSSA, methicillin-susceptible *Staphylococcus aureus*.

## Data Availability

The original contributions presented in this study are included in the article. Further inquiries can be directed to the corresponding author.
